# Baseline Uric Acid-to-HDL Cholesterol Ratio Predicts Peritoneal Membrane Failure in Peritoneal Dialysis Patients

**DOI:** 10.3390/jcm15062160

**Published:** 2026-03-12

**Authors:** Veysel Baran Tomar, Omer Faruk Akcay, Asil Demirezen, Taha Enes Cetin, Ayser Seda Hasdemir, Cansu Dagasan, Ozant Helvacı, Kadriye Altok, Yasemin Erten

**Affiliations:** 1 Department of Nephrology, Gazi University Faculty of Medicine, Ankara 06560, Turkey; omerfarukakcay@gazi.edu.tr (O.F.A.); demirezenasil@gazi.edu.tr (A.D.); tahaenes.cetin@gazi.edu.tr (T.E.C.); ayserseda.hasdemir@gazi.edu.tr (A.S.H.); ozanthelvaci@gazi.edu.tr (O.H.); kadriye@gazi.edu.tr (K.A.); yerten@gazi.edu.tr (Y.E.); 2 Department of Internal Medicine, Gazi University Faculty of Medicine, Ankara 06560, Turkey; cansu.dagasan@gazi.edu.tr

**Keywords:** peritoneal dialysis, peritoneal membrane failure, uric acid-to-HDL cholesterol ratio, biomarker, kidney transplantation, risk stratification

## Abstract

**Background/Objectives**: Peritoneal membrane failure remains a major limitation of peritoneal dialysis (PD). Systemic inflammation contributes to membrane dysfunction, yet simple predictive biomarkers are lacking. The uric acid-to-HDL cholesterol ratio (UHR) represents a novel integrative marker of metabolic-inflammatory burden, but its association with membrane failure has not been investigated. **Methods**: This retrospective cohort study included adult patients who initiated PD between 1997 and 2023. Baseline UHR was calculated from laboratory measurements obtained within the first three months after PD initiation. The primary outcome was peritoneal membrane failure, defined as permanent transfer to hemodialysis due to ultrafiltration failure, inadequate solute clearance, or progressive membrane dysfunction. Receiver operating characteristic, Kaplan–Meier, and Cox regression analyses were used to evaluate the association between UHR and membrane failure. **Results**: Among 214 patients, 62 (29%) developed membrane failure during follow-up. Baseline UHR was significantly higher in patients with membrane failure. A UHR cut-off value of 14 was identified for risk stratification. In multivariable Cox regression analysis, UHR >14 was independently associated with an increased risk of membrane failure (hazard ratio 1.836, 95% CI 1.040–3.241). A history of kidney transplantation prior to PD initiation also emerged as a strong independent predictor of membrane failure. **Conclusions**: Elevated baseline UHR is independently associated with peritoneal membrane failure in PD patients. As a simple and readily available biomarker, UHR may support early risk stratification and individualized management. Prospective multicenter studies are warranted to validate these findings.

## 1. Introduction

Peritoneal dialysis (PD) is a widely used kidney replacement modality for patients with end-stage kidney disease (ESKD), offering several advantages including preservation of residual kidney function (RKF), hemodynamic stability, and improved quality of life compared to hemodialysis (HD) [1]. Despite these benefits, long-term PD is often limited by technique failure, with peritoneal membrane-related complications representing a significant cause of treatment discontinuation. Previous studies have shown that approximately 20–30% of PD patients develop membrane failure over time, primarily due to ultrafiltration failure, inadequate solute clearance, or progressive membrane dysfunction [2,3]. Understanding the risk factors and developing reliable predictive biomarkers for membrane failure remain critical challenges in optimizing patient selection and long-term PD outcomes.

The pathophysiology of peritoneal membrane failure is a complex, multifactorial process involving chronic inflammation, oxidative stress, neoangiogenesis, and progressive fibrosis [4,5]. Prolonged exposure to glucose-based dialysate solutions, recurrent peritonitis episodes, and uremic toxins contribute to structural and functional alterations in the peritoneal membrane over time [6,7]. Recent comprehensive reviews have highlighted the multifactorial nature of PD discontinuation, emphasizing the importance of identifying early predictors of technique failure to optimize patient selection and long-term outcomes [8]. While several clinical and biochemical markers have been investigated as potential predictors of membrane dysfunction, including peritoneal effluent biomarkers [9], their clinical utility remains limited, and simple, accessible serum-based biomarkers for predicting membrane failure remain lacking. So, no single biomarker has been universally adopted in routine practice.

Emerging evidence suggests that systemic metabolic dysregulation may play a significant role in the development of peritoneal membrane complications. Uric acid, traditionally recognized for its role in gout and nephrolithiasis, has recently gained attention as a marker of oxidative stress, endothelial dysfunction, and systemic inflammation [10]. Elevated serum uric acid levels have been associated with increased cardiovascular morbidity and mortality in chronic kidney disease (CKD) patients, including those undergoing dialysis [11,12]. Conversely, high-density lipoprotein cholesterol (HDL-C) is well established as a protective factor against cardiovascular disease, possessing anti-inflammatory, antioxidant, and endothelial-protective properties [13]. The ratio of uric acid to HDL cholesterol (UHR) has emerged as a novel marker integrating both pro-oxidant and anti-inflammatory pathways, and has demonstrated predictive value for cardiovascular events and metabolic syndrome in various populations [14,15].

Recent studies have suggested a potential role of UHR as a prognostic marker in CKD populations [16]. Given that oxidative stress and inflammation are central mechanisms in peritoneal membrane deterioration, the biological rationale for evaluating UHR in PD patients seems compelling. A higher UHR may indicate an imbalance favoring pro-inflammatory and pro-oxidant processes, potentially predisposing patients to accelerated membrane damage. However, to our knowledge, no previous study has specifically examined the association between UHR and peritoneal membrane failure in PD patients.

Given the need for accessible, cost-effective biomarkers to identify patients at higher risk of membrane failure, we hypothesized that baseline UHR could serve as an independent predictor of peritoneal membrane failure. Therefore, we conducted this retrospective cohort study to evaluate the association between UHR and the risk of membrane failure in a well-characterized PD population and to assess its potential clinical utility for risk stratification.

## 2. Materials and Methods

### 2.1. Study Design and Population

This retrospective cohort study included adult patients who initiated PD and were followed at a single tertiary referral center between January 1997 and June 2025. Patients were eligible for inclusion if they were ≥18 years of age and had available baseline laboratory and clinical data obtained within the first three months after PD initiation. This early time window was chosen to capture the patients’ metabolic-inflammatory status prior to significant dialysis-related alterations, thereby reflecting true baseline characteristics for risk prediction. Of the 297 patients who initiated PD during the study period, 83 were excluded due to missing baseline serum uric acid and/or HDL cholesterol values, use of lipid-lowering and/or uric acid-lowering medications at the time of data collection, or clinical or laboratory evidence of active infection at baseline, resulting in a final cohort of 214 patients.

Follow-up time was calculated from the initiation of PD until the occurrence of membrane failure, death, kidney transplantation (KT), transfer to hemodialysis for non–membrane-related reasons, or end of follow-up, whichever occurred first.

### 2.2. Data Collection and Laboratory Measurements

Demographic and clinical data, including age, sex, body mass index (BMI), comorbidities [diabetes mellitus (DM), hypertension (HT), coronary artery disease (CAD), and KT history], and smoking status, were retrieved from medical records. Baseline laboratory values, including serum glucose, hemoglobin, albumin, parathyroid hormone (PTH), uric acid, triglyceride, low-density lipoprotein cholesterol (LDL-C), and HDL-C, were obtained during routine outpatient follow-up within the first three months after PD initiation.

The UHR was calculated as the ratio of serum uric acid (mg/dL) to HDL-C (mg/dL), multiplied by 100 [17].

The Triglyceride–Glucose (TyG) index was calculated using the formula: ln [fasting triglyceride (mg/dL) × fasting glucose (mg/dL)/2] [18].

### 2.3. Peritoneal Dialysis Characteristics

The baseline peritoneal equilibration test (PET) was performed approximately 4–6 weeks after initiation of peritoneal dialysis, using a standard 4 h dwell with a 2.5% glucose solution. Patients were categorized according to the dialysate-to-plasma creatinine ratio. PD modality [continuous ambulatory peritoneal dialysis (CAPD) or automated peritoneal dialysis (APD)] and baseline peritoneal transport characteristics were recorded. Information on assisted PD, duration of PD, and peritonitis episodes during follow-up was collected. RKF was assessed based on daily urine volume. A urine output of ≥100 mL/day was used to define non-anuric status as a surrogate indicator of preserved RKF, consistent with NKF-KDOQI guidance, which states that urine output ≥100 mL/day should be considered significant in dialysis patients, whereas those with <100 mL/day were classified as anuric and considered to have no RKF [19].

Mandatory PD was defined as initiation of PD when HD was not feasible due to lack or failure of suitable vascular access. Elective PD referred to cases in which PD was chosen as the preferred renal replacement modality among available options.

### 2.4. Outcome Definition

The primary outcome was peritoneal membrane failure, defined as permanent transfer to HD due to ultrafiltration failure, inadequate solute clearance, or progressive peritoneal membrane dysfunction. Transfers to HD for reasons unrelated to peritoneal membrane dysfunction (e.g., patient preference, catheter-related complications, or social reasons), and directly attributable to acute peritonitis, were not considered events.

### 2.5. Statistical Analysis

Statistical analyses were performed using SPSS version 25.0 (IBM Corp., Armonk, NY, USA). The normality of the data distribution was assessed using the Kolmogorov–Smirnov test. Continuous variables were expressed as mean ± standard deviation (SD) or median [interquartile range (IQR)], depending on distribution, while categorical variables were presented as frequencies and percentages. Comparison of baseline characteristics between groups (Membrane Failure vs. No Failure) was performed using Student’s *t*-test or Mann–Whitney U test for continuous variables, and the Chi-square test for categorical variables. Correlations between UHR and other metabolic parameters (BMI, TyG index) were evaluated using Spearman’s rank correlation analysis. To determine UHR’s discriminatory ability for predicting membrane failure, Receiver Operating Characteristic (ROC) curve analysis was performed, and the Area Under the Curve (AUC) was calculated. An optimal cut-off value was determined to categorize patients into high- and low-UHR groups. Survival curves were generated using the Kaplan–Meier method, and differences between groups were compared using the Log-rank test. Univariate and multivariate Cox proportional hazards regression models were used to identify independent predictors of peritoneal membrane failure. Variables with a *p*-value <0.1 in the univariate analysis or clinically significant factors were included in the multivariate model. To prevent potential multicollinearity, serum uric acid and HDL cholesterol were not entered simultaneously with UHR into the multivariable regression models. A *p*-value of <0.05 was considered statistically significant.

## 3. Results

### 3.1. Baseline Characteristics of the Study Population

A total of 214 PD patients were included in this study. During the follow-up period, peritoneal membrane failure developed in 62 (29%) patients. The baseline demographic and clinical characteristics of the patients, stratified by the development of membrane failure, are presented in Table 1. There were no statistically significant differences between the groups regarding age, sex, BMI, and the prevalence of comorbidities such as DM and HT. However, the median UHR was significantly higher in patients who developed membrane failure compared to those who did not (17.3 [IQR: 13.7–21.6] vs. 14.0 [IQR: 10.3–18.8], *p* = 0.001). Additionally, patients with membrane failure had a significantly longer PD duration (38.3 months vs. 27.7 months, *p* = 0.036) and a higher prevalence of CAPD modality (83.9% vs. 65.1%, *p* = 0.006).

### 3.2. Correlations Between UHR and Clinical Parameters

**Figure 1:**
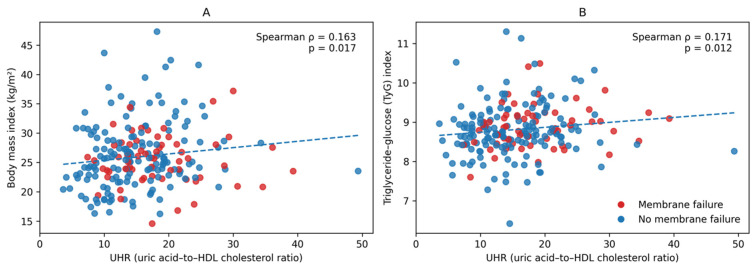
Correlation between uric acid-to-HDL cholesterol ratio (UHR) and metabolic parameters. (**A**) Scatter plot showing the relationship between UHR and body mass index (BMI). (**B**) Scatter plot showing the relationship between UHR and the triglyceride–glucose (TyG) index. Red dots indicate patients who developed peritoneal membrane failure, while blue dots represent patients without membrane failure. Dashed lines indicate the fitted trend lines.

Spearman correlation analysis was performed to evaluate the relationship between UHR and metabolic parameters. As shown in Figure 1, there was a weak but statistically significant positive correlation between UHR and BMI (r = 0.163, *p* = 0.017). Similarly, a positive correlation was observed between UHR and TyG index (r = 0.171, *p* = 0.012), suggesting an association between higher UHR levels and markers of metabolic dysregulation.

### 3.3. Diagnostic Performance of UHR for Predicting Membrane Failure

**Figure 2:**
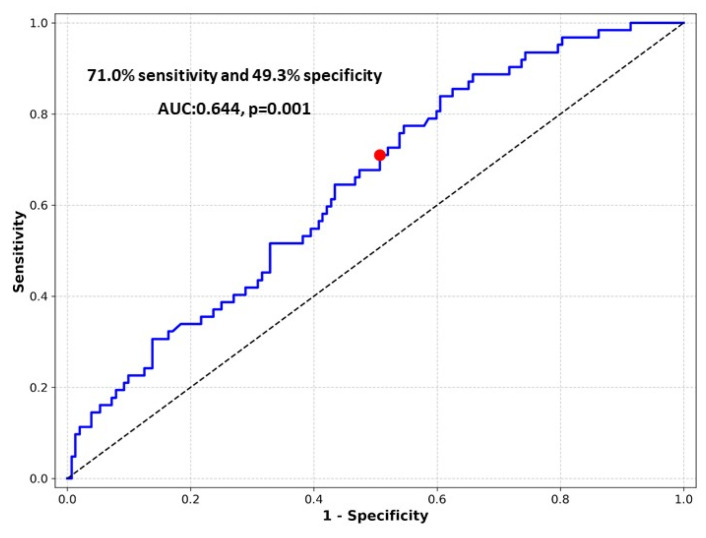
Receiver operating characteristic (ROC) curve of UHR for predicting peritoneal membrane failure. The blue line represents the ROC curve, the red dot indicates the optimal cutoff point, and the dotted diagonal line represents the reference line of no discrimination.

The predictive value of UHR for peritoneal membrane failure was assessed using ROC curve analysis (Figure 2). The AUC for UHR was 0.644 (95% CI: 0.566–0.722, *p* = 0.001), indicating modest but statistically significant discriminative ability. Based on ROC curve analysis, an optimal UHR cut-off value of 14 was identified using the Youden index to stratify patients for subsequent survival analyses. At this threshold, the sensitivity and specificity for predicting membrane failure were 71.0% and 49.3%, respectively.

### 3.4. Survival Analysis and Independent Predictors of Membrane Failure

**Figure 3:**
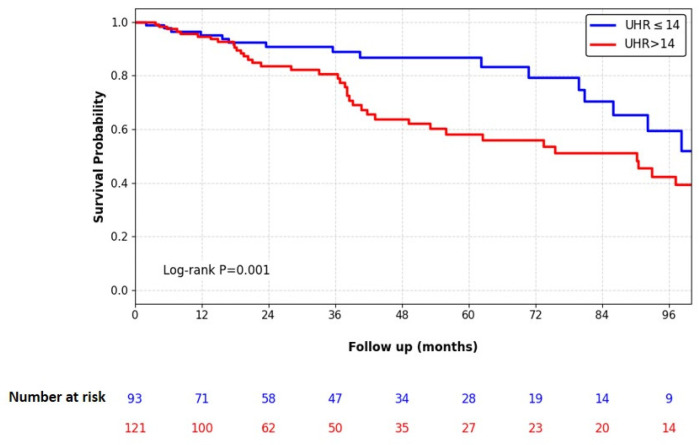
Kaplan–Meier analysis of peritoneal membrane survival according to baseline UHR.

Kaplan–Meier survival analysis demonstrated that patients with a high UHR (>14) had a significantly lower membrane survival rate compared to those with a lower UHR (Log-rank *p* = 0.001), as illustrated in Figure 3. To identify independent predictors of peritoneal membrane failure, univariate and multivariate Cox proportional hazards regression analyses were conducted (Table 2). In the univariate analysis, male sex (HR: 1.748, *p* = 0.033), history of KT before PD (HR: 3.677, *p* = 0.003), and UHR > 14 (HR: 1.971, *p* = 0.016) were associated with an increased risk of membrane failure. In the multivariate model, adjusted for confounding factors, UHR > 14 remained an independent predictor of membrane failure, increasing the risk by approximately 1.8-fold (HR: 1.836, 95% CI: 1.040–3.241, *p* = 0.036). A history of KT before PD was also identified as a strong independent predictor (HR: 3.971, 95% CI: 1.668–9.455, *p* = 0.002). Male sex, however, lost its statistical significance in the multivariate analysis (*p* = 0.100).

## 4. Discussion

This retrospective cohort study provides the first evidence that the UHR is independently associated with peritoneal membrane failure in patients undergoing peritoneal dialysis. We demonstrated that an elevated baseline UHR was associated with an approximately 1.8-fold increased risk of membrane failure, even after adjustment for relevant clinical and dialysis-related factors. In addition, a history of kidney transplantation before PD initiation emerged as a strong independent predictor of membrane failure. Together, these findings suggest that UHR is a clinically accessible marker reflecting systemic metabolic–inflammatory burden and may aid in early risk stratification and individualized management of PD patients.

UHR captures the interplay between two complementary biological pathways: pro-oxidative inflammatory activity driven by uric acid and the anti-inflammatory, endothelial-protective functions of HDL-C [13,20]. In this context, elevated uric acid may exacerbate endothelial dysfunction and promote inflammatory activation. In contrast, HDL-C in uremic states is often structurally and functionally altered, leading to a loss of its vasculoprotective properties [21]. Concurrently, chronic exposure to glucose-based dialysate solutions and the uremic milieu can further intensify oxidative stress and systemic inflammation, thereby contributing to progressive peritoneal membrane injury [6,7]. Taken together, our findings suggest that UHR represents a clinically accessible composite marker of metabolic–inflammatory imbalance, linking systemic stress to subsequent peritoneal membrane failure in patients undergoing PD.

In our cohort, higher UHR values were significantly associated with subsequent peritoneal membrane failure, demonstrating a modest but statistically significant discriminatory capacity (AUC = 0.644). Although this level of discrimination reflects fair rather than excellent predictive performance, peritoneal membrane failure is a complex, multifactorial outcome unlikely to be captured by any single biomarker. Notably, other commonly assessed biochemical markers reflecting nutritional and metabolic status, such as serum albumin, CRP, and the TyG index, showed no significant differences between patients with and without membrane failure and were therefore not included in additional predictive modeling.

In contrast, the positive correlations observed between UHR and both BMI and the TyG index provide supporting evidence that elevated UHR levels may reflect an underlying state of metabolic dysregulation. These associations support the concept that UHR functions not only as a standalone predictor but also as a surrogate marker of systemic metabolic–inflammatory burden that may contribute to progressive peritoneal membrane dysfunction. Notably, despite its modest discriminatory performance, UHR >14 remained an independent predictor of membrane failure in multivariable Cox regression analysis, reinforcing its potential as a simple, cost-effective biomarker in clinical practice, particularly in resource-limited settings. When interpreted alongside established clinical parameters, such as peritoneal transport characteristics and RKF, UHR may contribute to a more comprehensive assessment of long-term PD outcomes.

We observed a higher prevalence of CAPD among patients who developed peritoneal membrane failure compared with those treated with APD. This difference may reflect distinct patterns of dialysate exposure, glucose load, and local peritoneal stress between PD modalities. CAPD is characterized by continuous exposure of the peritoneal membrane to glucose-based dialysate, potentially leading to greater cumulative glucose burden and sustained inflammatory activation. In contrast, APD, with shorter dwell times and cycler-assisted exchanges, may allow intermittent periods of relative membrane recovery [22]. However, this association did not persist in multivariable analysis, suggesting that PD modality itself is unlikely to be an independent determinant of membrane failure. Instead, the observed unadjusted difference may be influenced by patient selection factors, as APD is more frequently prescribed to younger, more active patients or those with greater social support and fewer comorbidities, who may inherently carry a lower baseline risk for membrane-related complications. The absence of an independent association between peritonitis episodes and membrane failure in our analyses may reflect heterogeneity in peritonitis severity, frequency, and timing, which could not be captured in sufficient detail for analytic adjustment. Accordingly, these findings should be interpreted cautiously and regarded as hypothesis-generating, warranting confirmation in prospective studies with comprehensive adjustment for clinical, metabolic, and socioeconomic factors.

An additional and notable finding of our study was the strong association between a history of KT prior to PD initiation and subsequent peritoneal membrane failure. These observations are in line with previous registry-based studies demonstrating inferior PD outcomes among patients returning to dialysis after graft failure [23,24]. Patients with failed KT may experience persistent immune dysregulation and chronic inflammation related to previous allograft exposure and immunosuppressive therapy [25]. Accordingly, this association may reflect sustained systemic inflammatory activity following graft loss. Surgical interventions and chronic alloimmune stimulation may further contribute to ongoing inflammatory and metabolic stress, thereby predisposing the peritoneal membrane to accelerated injury. Furthermore, patients with KT prior to PD initiation often have longer cumulative exposure to uremia and dialysis therapies, potentially increasing baseline susceptibility to membrane dysfunction. Together, these mechanisms may explain the substantially increased risk of membrane failure observed in this subgroup and underscore the importance of incorporating transplant history into risk stratification and modality selection when considering PD after graft loss [26].

Despite these strengths, several limitations warrant consideration. The retrospective, single-center design may limit the generalizability of the findings, and changes in PD practice over the long study period could not be fully accounted for in the analyses. Moreover, the optimal UHR cutoff identified in this study requires external validation in independent cohorts. Finally, the observational nature of the study precludes causal inference.

## 5. Conclusions

In this retrospective study, an elevated baseline UHR was identified as an independent predictor of peritoneal membrane failure in patients undergoing PD. Its association with adverse outcomes supports UHR as a potential integrative biomarker that reflects the combined burden of inflammation, oxidative stress, and metabolic dysregulation. Given its simplicity, cost-effectiveness, and accessibility, UHR may serve as a practical tool for early risk stratification in clinical practice. A history of KT prior to PD initiation was also identified as a strong independent risk factor for membrane failure, underscoring the need for careful assessment when selecting dialysis modality after transplant failure. Further prospective, multicenter studies are needed to validate the prognostic utility of UHR, establish standardized cutoff values, and explore its potential role within composite risk assessment frameworks for long-term PD outcomes. Integrating UHR with established clinical parameters, such as peritoneal transport characteristics and RKF, may enable a more comprehensive assessment of long-term PD suitability.

## Figures and Tables

**Table 1:** Baseline characteristics of peritoneal dialysis patients according to peritoneal membrane failure.

	Total *n* = 214	Non-Failure *n* = 152 (71%)	Failure *n* = 62 (29%)	*p* Value
Sex (female)	101 (47.2)	75 (49.3)	26 (41.9)	0.325
Age (years)	48.2 ± 17.3	49.4 ± 17.2	45.2 ± 17.5	0.112
BMI	25.3 (22.5–28.7)	25.2 (22.5–29.3)	25.5 (22.7–28.0)	0.288
DM	58 (27.1)	46 (30.3)	12 (19.4)	0.103
HT	182 (85.0)	131 (86.2)	51 (82.3)	0.465
CAD	39 (18.2)	28 (18.4)	11 (17.7)	0.907
Smoking	34 (15.9)	25 (16.4)	9 (14.5)	0.726
KT history before PD	12 (5.6)	6 (3.9)	6 (9.7)	0.098
PD choice Mandatory	20 (9.3)	15 (9.9)	5 (8.1)	0.681
Previous hernia history	35 (16.4)	23 (15.1)	12 (19.4)	0.449
Laboratory values Hemoglobin (g/dL) Albumin (g/dL) CRP (mg/dL) PTH (pg/mL) LDL-Cholesterol	11.0 ± 1.8 3.8 (3.4–4.1) 3.0 (3.0–9.6) 276 (173–529) 106 (85–135)	11.1 ± 1.7 3.9 (3.5–4.1) 4.1 (3.0–10.2) 302 (174–553) 107 (83–135)	10.6 ± 2.0 3.7 (3.3–4.1) 3.0 (3.0–7.0) 247 (169–449) 105 (89–134)	0.176 0.108 0.349 0.372 0.911
UHR	14.8 (11.1–19.3)	14.0 (10.3–18.8)	17.3 (13.7–21.6)	**0.001**
TyG index	8.8 (8.5–9.2)	8.7 (8.4–9.1)	8.9 (8.5–9.2)	0.192
Baseline RRF (yes)	199 (93.9)	143 (94.1)	56 (90.3)	0.329
Baseline weekly Kt/V	2.25 ± 0.76	2.31 ± 0.76	2.12 ± 0.76	0.081
PET category *Low—Low average* *High average—High*	112 (52.3) 102 (47.7)	86 (56.6) 66 (43.4)	26 (41.9) 36 (58.1)	0.052
PD modality *CAPD* *APD*	151 (70.6) 63 (29.4)	99 (65.1) 53 (34.9)	52 (83.9) 10 (16.1)	**0.006**
Assisted PD	22 (10.3)	15 (9.9)	7 (11.3)	0.756
Any peritonitis episodes at follow-up, *n* (%)	74 (34.6)	52 (34.2)	22 (35.5)	0.859
PD duration (months)	31.0 (15.0–61.5)	27.7 (13.8–54.1)	38.3 (17.8–80.8)	**0.036**

APD: Automated Peritoneal Dialysis, BMI: Body Mass Index, CAD: Coronary Artery Disease, CAPD: Continuous Ambulatory Peritoneal Dialysis, CRP: C-reactive Protein, DM: Diabetes Mellitus, HT: Hypertension, KT: Kidney Transplantation, PD: Peritoneal Dialysis, LDL: Low-density lipoprotein, PET: Peritoneal Equilibration Test, PTH: Parathyroid Hormone, RRF: Residual Renal Function, TyG index: Triglyceride–glucose index, UHR: Uric acid-to-HDL cholesterol ratio. *p*-value shown in bold indicate statistical significance at the *p* < 0.05 level.

**Table 2:** Univariate and Multivariate Cox regression analysis for peritoneal membrane failure.

	Univariate, HR (%95 CI)	*p* Value	Multivariate, HR (%95 CI)	*p* Value
Age	1.002 (0.987–1.018)	0.771		
Male sex	1.748 (1.045–2.924)	** 0.033 **	1.562 (0.918–2.658)	0.100
BMI	0.994 (0.944–1.046)	0.815		
DM	1.371 (0.714–2.631)	0.343		
Transplantation before PD	3.677 (1.561–8.662)	**0.003**	3.971 (1.668–9.455)	**0.002**
PD mandatory	0.689 (0.275–1.729)	0.428		
RRF (yes)	1.006 (0.430–2.355)	0.988		
PD modality (APD)	1.258 (0.611–2.590)	0.533		
Baseline PET category (High/High-average)	1.533 (0.912–2.576)	0.107		
Any peritonitis episodes	0.758 (0.443–1.297)	0.312		
TyG index	1.369 (0.911–2.056)	0.131		
UHR > 14	1.971 (1.134–3.424)	** 0.016 **	1.836 (1.040–3.241)	**0.036**

APD: Automated Peritoneal Dialysis, BMI: Body Mass Index, DM: Diabetes Mellitus, PD: Peritoneal Dialysis, PET: Peritoneal Equilibration Test, RRF: Residual Renal Function, TyG index: Triglyceride–glucose index, UHR: Uric acid-to-HDL cholesterol ratio. *p*-value shown in bold indicate statistical significance at the *p* < 0.05 level.

## Data Availability

The data presented in this study are available on request from the corresponding author due to institutional ethical approval and data protection regulations.
